# Siblings’ Risk of Adenoid Hypertrophy: A Cohort Study in Children

**DOI:** 10.3390/ijerph20042910

**Published:** 2023-02-07

**Authors:** Aleksander Zwierz, Krzysztof Domagalski, Krystyna Masna, Paweł Burduk

**Affiliations:** 1 Department of Otolaryngology, Phoniatrics and Audiology, Faculty of Health Sciences, Ludwik Rydygier Collegium Medicum, Nicolaus Copernicus University, 85-168 Bydgoszcz, Poland; 2 Department of Immunology, Faculty of Biological and Veterinary Sciences, Nicolaus Copernicus University, 87-100 Torun, Poland

**Keywords:** adenoid hypertrophy, AH, siblings, flexible nasopharyngoscopy, adenoid symptoms

## Abstract

Background: The aim of this study was to compare adenoid size in preschool-age siblings using flexible nasopharyngoscopy examination (FNE) when they reach the same age. The occurrence of adenoid symptoms in these patients was also analyzed. This study was conducted to analyze the adenoid size in siblings when they reach the same age and substantiate a correlation between adenoid hypertrophy (AH) and adenoid symptoms. Methods: We analyzed and reported on the symptoms, ENT examination results, and FNE of 49 pairs of siblings who were examined at the same age. Results: There was a strong association in adenoid size between siblings when they are at a similar age (r = 0.673, *p* < 0.001). Second-born children whose older sibling had III^o^ AH (A/C ratio > 65%) had a risk of III^o^ AH 26 times greater than patients whose older sibling did not have III^o^ AH (OR = 26.30, 95% CI = 2.82–245.54). Over 90% of snoring children whose siblings had confirmed III^o^ AH would develop III^o^ AH by the time they reach the same age. Second-born children in whom snoring occurs and whose older siblings have a III^o^ AH have about a 46 times higher risk of III^o^ AH compared to patients who did not meet these two conditions (*p* < 0.001, OR = 46.67, 95% CI = 8.37–260.30). Conclusions: A significant familial correlation between adenoid size in siblings when they reach the same age was shown. If the older sibling has a confirmed overgrown adenoid (III^o^ AH) and their younger sibling presents adenoid symptoms, particularly snoring, it is highly probable that they will also have an overgrown adenoid.

## 1. Introduction

Adenoid hypertrophy (AH) is one of the most common diseases among preschool children, usually associated with adenoid symptoms, such as mouth breathing, persistent rhinitis, snoring, and a nasal voice [1]. If the disease presents in an older child of a family, parents will often suspect that the same symptoms described above will resurface later in a younger sibling in the family. A common question is whether this problem is familial, especially if the parents also underwent or were considered as a child for adenoidectomy. In 1980, Katznelson and Gross first confirmed a significantly higher incidence of prior tonsillectomy and adenoidectomy performed on an analyzed group of siblings and parents than controls [2]. However, all later performed analyses and surgical procedures were based on presented adenoid symptoms, not on a true measurement of adenoid size. Thus, it is difficult to compare parents and their children, because diagnostic techniques have changed and improved over the years. Nowadays, flexible nasopharyngoscopy examination (FNE) seems the gold standard, not only for assessing adenoid size but also for checking the mucus coverage of the adenoid [3].

It is commonly believed that the adenoid undergoes hypertrophy during childhood, and eventually, involution in adulthood [4]. Over the years, several longitudinal studies, Handelman and Osborne (1976), Ishida et al. (2018), and Yamada et al. (2021), have assessed the size of the adenoid using lateral cephalometric radiography [5,6,7]. Yamada showed that an overgrowth of adenoids occurred in preschool children, but there were no significant changes in the adenoid size from 8–12 years of age [7]. A previous study based on flexible endoscopic examinations revealed that adenoid involution proceeds rather slowly; only 7.9% of preschool children (aged 3–7 years) underwent a change in the adenoid size by >15% on the adenoid to choana (A/C) ratio over one year of observation, 21.6% over a period of 2 years, and 45% over a period of 3 years [8]. The growth and development patterns of the nasopharyngeal lymphoid tissues vary for each individual; accordingly, we believe that in studies comparing the adenoid size in a pair of siblings, the permissible age difference should not exceed 12 months.

This study aimed to compare the adenoid size of siblings who were raised in the same household. In most cases, the pairs of children studied were raised with exposure to the same environmental factors, such as cigarette smoke, pollution, and mold allergens, which are considered risk factors for the development of AH [9,10]. Tobacco smoke exposure has been particularly reported as a risk factor for AH [10]; however, nowadays, numerous preventive campaigns have been conducted in our country, and parents are abundantly aware of the risks associated with cigarette smoke exposure in children. Therefore, there is a common practice of avoiding smoking in rooms where children reside. Nevertheless, parents who violate this rule will usually not admit it. Furthermore, the city and rural living conditions may vary in terms of air pollution and allergen exposure, so we analyzed the influence of area of residence on AH. Notably, the duration of breastfeeding is a potential factor distinguishing between babies living in one environment, and breastfeeding has been indicated as a risk factor for snoring and obstructive sleep apnea syndrome (OSAS) [11,12,13]. Xu et al. stated this correlation and highlighted the need for further investigations to confirm the relationship between breastfeeding and OSAS and the mechanisms underlying it [11]. Chng et al. suggest that breastfeeding independently increases the risk of snoring and possible obstructive sleep apnea syndrome [12]. In another study, Montomery-Downs et al. indicated that OSAS severity is reduced in association with breastfeeding, but it should not be interpreted to suggest that breastfeeding entirely prevents the development of sleep disorder breathing [13]. Presumably, AH may be operational in this mechanism; however, to the best of our knowledge, no study has confirmed a relationship between breastfeeding and AH. Moreover, no genetic factors associated with AH have been discovered so far to demonstrate a similarity in the adenoid size of siblings and justify further research.

FNE is a common procedure to evaluate adenoid size, and this study used FNE to compare adenoid sizes in siblings when they reach the same age. This study was conducted to analyze the adenoid size in siblings when they reach the same age and substantiate a correlation between AH and adenoid symptoms. 

## 2. Materials and Methods

### 2.1. Study Population 

We retrospectively analyzed a group of 1247 preschool children (3–7 year of age) who visited a medical outpatient ear, nose, and throat (ENT) clinic with symptoms suggestive of chronic AH between 2016 and 2021. We searched the medical history of all preschool children admitted to the ENT outpatient clinic. Then, 82 pairs of siblings were selected. We included in the study each pair of children if they were examined in the ENT outpatient clinic at around the same age, where the permissible age difference should not exceed 12 months. We then called their caretakers to confirm if the siblings had the same parents. Exclusion criteria from the study were: children brought up in a common household who have the same last name but different parents, craniofacial anomalies, such as cleft lip/cleft palate; genetic diseases (Down Syndrome); septal nasal deviation; nasal polyp or inferior turbinate hypertrophy; active upper respiratory infection within 2 weeks of enrolling in the study; or previously performed adenoidectomy. In the end, 49 pairs of siblings qualified for participation in the study.

The initial assessment of each patient after study enrollment included a parental questionnaire concerning recurrent upper respiratory infections, defined as a frequent runny nose, pharyngitis, or a cough [14]. We also analyzed the symptoms of rhinitis—at least two nasal symptoms: rhinorrhea, blockage, sneezing, or itching and snoring—defined as persistent, occasional, or non-existent [15]. All children performed an ENT physical examination, flexible fiberoptic rhinoscopy, and tympanometry. 

Additionally, we analyzed whether residing in the city or rural regions affects the adenoid size. We divided the children into two groups: those living in the city (population: 170,000–340,000 citizens), and those living in the countryside.

Seasons may influence adenoid mucus coverage and tympanometry type [3]. To avoid any seasonal influence on the obtained results and compare better the sibling population from this study, we divided the year into two main seasons, winter and summer, and we considered the cut-off temperature to be 10 °C and also analyzed seasons of performed examination.

### 2.2. Endoscopy

Each child underwent flexible endoscopic examinations using common nasal meatus, performed by one pediatric otorhinolaryngologist (A.Z.) using the Karl Storz Tele Pack endoscopic system, which was equipped with a flexible nasopharyngoscope (2.8-mm outer diameter and 300-mm length). The percentages of obturation (adenoid-to-choanae ratio in percentage-A/C ratio) of the choanae and mucus coverage of the adenoids were analyzed based on videoendoscopy with the freeze-frame option. Choanal obstructions were assessed with an accuracy of up to 5%. For a better statistical assessment, patients were divided into groups for which we used the 3-degree Bolesławska scale, particularly the part concerning adenoid size in relation to the nasopharynx [16]. All recorded videos of the nasopharynx were coded and blindly analyzed. The percentage of choanal obstruction by the adenoid was measured and compared between siblings. Adenoid size and mucus coverage recorded on the endoscopic system were compared by a second doctor (K.M.). If there was a discrepancy in the assessment, the score was reassessed by a third ENT doctor (P.B.).

The difference in adenoid size between each sibling pair was considered a percentage difference in the amount of nasopharyngeal obstruction by the adenoid. In addition, we used the previous proposed scale to assess the mucus coverage of the adenoid, called the Mucus of Adenoid Scale by Nasopharyngoscopy Assessment (MASNA), which describes the amount of mucus covering the adenoid on a four-point scale (0, no mucus; 1, residue of clear watery mucus; 2, some amount of dense mucus; 3, copious thick dense mucus) [3]. 

### 2.3. Tympanometry

An otoscopic examination was performed, and if needed, the external auditory canal was cleaned. In addition, tympanometry was performed using the GSI 39 Auto Tymp^TM^ by Grason-Stadler. The middle ear effusion in each ear was analyzed by tympanometry measurement, and tympanogram graphs were generated. The results were classified using the classification system for tympanograms developed by Liden and Jerger [17,18]. The sequence of saved tympanograms for each patient ear was right, left. We posit that type-B tympanograms produced the worst result, type-C, significant negative pressure in the middle ear, was worse, and type-A, normal middle ear status, was good. For a further statistical analysis, we divided the children into three groups, considering the worse tympanogram result for each child: Group A children with type-A tympanogram in both ears (AA), group C children with tympanogram C (CC, AC, and CA), and group B children with tympanogram B (BB, BC, CB, AB, and BA). 

### 2.4. Ethics

Ethical approval for this study was obtained by the ethics committee of Nicolaus Copernicus University (KB 559/2021). 

### 2.5. Statistical Analysis

We used descriptive statistics to summarize and describe the variables for the study group. We summarized quantitative variables, such as age and adenoid size, based on their means ± standard deviations (SDs). For the categorical variables, we used frequency counts and percentages. To determine differences between variables, statistical significance was estimated using the Chi-square method or Fisher’s exact test for categorical variables and the Student’s t-test or ANOVA for quantitative variables. Associations between variables were analyzed using Pearson’s correlation. 

Variables significantly related to adenoid size in a univariate analysis were included in the linear and logistic regression analyses. The linear regression analysis assessed variables of significance for the prediction of adenoid size (A/C ratio, %) volatility in the whole study group. In our linear regression analysis, variables such as recurrent upper respiratory tract infections, rhinitis, snoring, adenoid mucus coverage, and type of tympanogram were assigned appropriate values (for recurrent upper respiratory tract infections (rURTI), rhinitis, snoring: 0, symptom not present; 1, symptom present; for adenoid mucus coverage: from 0 to 4 according MASNA scale; for tympanogram type: 0–A, 1–C, 2–B).

To check for any differences between the pairs of analyzed siblings, the children were divided into two groups: the first including the first examined child from the pair, usually the older of the siblings, and the second including the second examined child, usually the younger. If large families were analyzed, only one pair of siblings from a given family was preferred. We selected the sibling pair with the smallest age difference at the time of examinations.

To analyze the associations between a significant increase in adenoid size (AH) in the second-born child and clinical factors, such as recurrent upper respiratory tract infections, rhinitis, snoring, adenoid mucus coverage, and tympanogram type as categorical variables, a logistic regression analysis was performed. III^o^ AH was defined as an A/C ratio of >65%, based on the Bolesławska scale, where 65% is the cut-off point between II^o^ and III^o^ AH. To predict an A/C ratio of >65% in second-born children, we conducted two separate assessments of: (1) second-born factors and (2) second-born factors and first-born adenoid size. Odds ratios (ORs) and 95% confidence intervals (95% CIs) were also calculated for the considered clinical variables in the regression models.

For all these tests, two-tailed *p*-values were used, and differences at the level of *p* < 0.05 were considered significant. All statistical analyses were performed using the SPSS (Statistical Package for the Social Sciences version 26, Armonk, NY, USA) software.

## 3. Results

The mean age of the first examined child group was 5.0 years (SD = 2.2), and that of the second examined child group was 5.1 (SD = 2.2). The mean adenoid size as an A/C ratio for the first sample was 63%, and it was 59% for the second. In total, 71.4% of parents reported rURTI in the first group of siblings and 51% in the second. Rhinitis was present in 77.6% of children from the first group and in 65.3% from the second group. Persistent and occasional snoring were present in, respectively, 36.7% and 28.6% of children from the first group and 34.7% and 26.5% from the second group. Mucous coverage of the adenoid according to MASNA scale grades 0 to 3 was, respectively, 30.6%, 44.9%, 18.4%, and 6.1% in the first group and 28.6%, 38.8%, 24.5%, and 8.2% in the second group. Analyzing the tympanometry results, we found 53.1% type-A tympanograms, 20.4% type-C tympanograms, and 26.5% type-B tympanograms in the first group and 67.3% type-A tympanograms, 12.2% type-C tympanograms, and 20.4% type-B tympanograms in the second group. Comparing examinations in thermal seasons, 42.9% of children were examined in the summer and 57.1% in the winter in the first group and 55.1% in the summer and 44.9% in the winter in the second group. No differences between groups were found in terms of the analyzed data, except rURTI. All presented data are included in Table 1.

**Figure 1:**
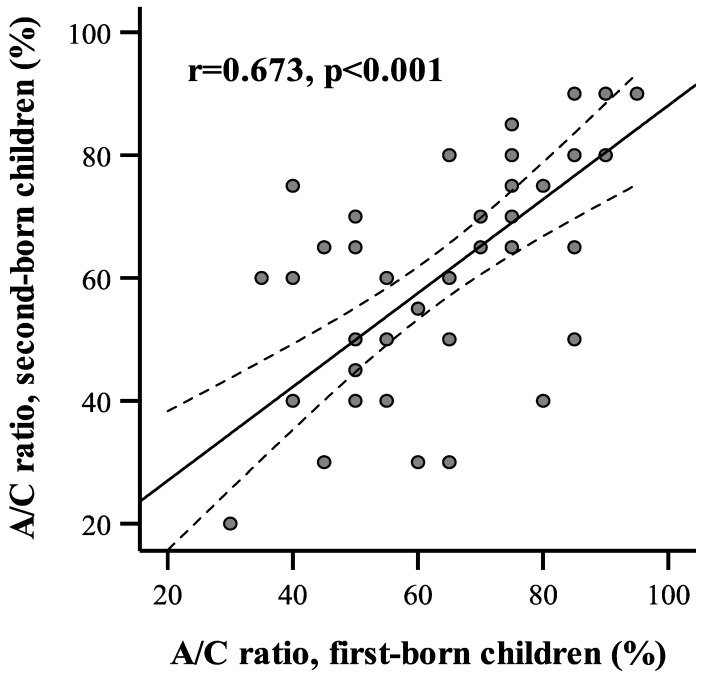
A/C ratio correlation between the first- and second-born children in the family.

An association between adenoid size in siblings was determined by Pearson’s correlation analysis. The correlation coefficient between adenoid size on the A/C ratio between the first- and the second-born siblings showed a strong positive association (r = 0.673, *p* < 0.001 (Figure 1). 

**Figure 2:**
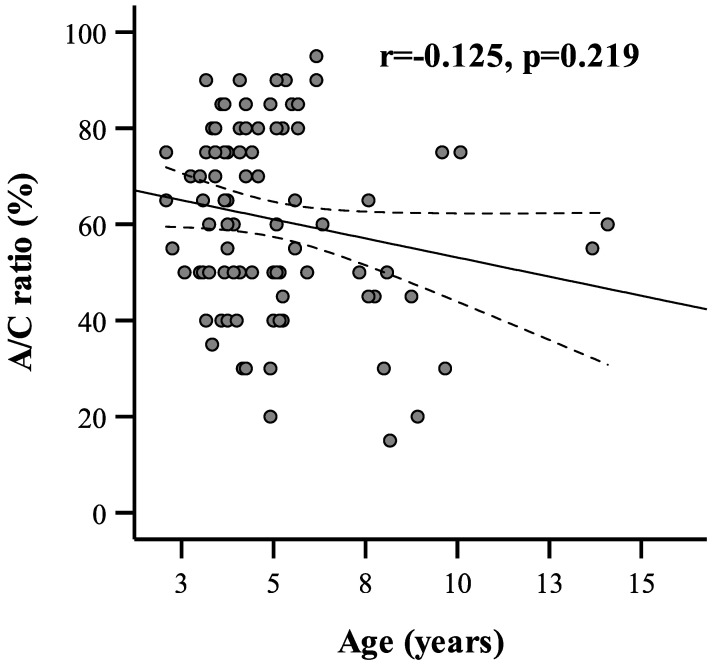
Correlation between A/C ratio and age of study population (*n* = 98).

In the next analysis step, the relationships between demographic or clinical factors and adenoid size were analyzed for the entire group of children (Table 2). Statistically significant differences in A/C ratios were obtained for rURTI (*p* < 0.001), rhinitis (*p* < 0.001), snoring (*p* < 0.001), tympanometry type (*p* < 0.001), and adenoid mucus coverage (*p* = 0.002). Patients with rURTI and rhinitis, snoring, impaired tympanogram, or high adenoid mucus coverage according to the MASNA scale had an increased A/C ratio. There were no associations between adenoid size and sex and seasonality. Moreover, we analyzed adenoid size change according to age for the whole patient sample (Figure 2). A linear correlation analysis showed no significant correlation between age and adenoid size (r = −0.125, *p* = 0.219). We also analyzed the living conditions (in the city or countryside) as an environmental factor influencing the adenoid size. No significant correlation between adenoid size in children living in city and countryside was found.

Variables significantly related to adenoid size in the univariate analysis were included in the linear and logistic regression analyses to identify independent prognostic factors useful in assessing adenoid size. Linear regression analysis for predicting the adenoid size variance in the whole study group revealed that rURTI, snoring, adenoid mucus coverage, and tympanogram type impact the assessment of adenoid size, but not rhinitis (Table 3). Interestingly, the most important aspect is whether the patient is snoring (β = 0.329), followed by the tympanogram type (β = 0.269).

The main analysis in this study focused on assessing the significance of clinical factors in the prognosis of III^o^ AH in siblings. For this, logistic regression analyses for the detection of III^o^ AH (A/C ratio > 65%) in second-born children were performed. Using second-born child factors, logistic regression analyses showed importance only for snoring (*p* = 0.006) and tympanogram type (*p* = 0.029; Table 4). Patients diagnosed with snoring have more than a 6-fold greater risk of III^o^ AH (OR = 6.23, 95% CI = 1.7–22.70) compared to patients who do not snore. Likewise, the presence of an ear impairment demonstrated by type-B or -C tympanogram relates to an over 6-fold increase in the risk of III^o^ AH (OR = 6.30, 95% CI = 1.20–32.99).

As shown, there was a strong association in adenoid size between siblings at a similar age. Therefore, we subsequently analyzed the importance of adenoid size in older siblings in relation to assessing adenoid size in younger siblings using logistic regression analysis. For this analysis, the adenoid size of the first-born child was categorized as the binary clinical variable (A/C ratio ≤ 65% vs. >65%). According to the assumed criterion, an A/C ratio of >65% was detected in 20 first-born children (40.8%). The logistic regression analysis showed that the assumed variable had the strongest significant effect on the prediction of III^o^ AH (*p* = 0.004; Table 4). The obtained estimates indicate that second-born children whose older siblings had III^o^ AH had more than a 26-fold greater risk of III^o^ AH compared to patients whose older sibling did not have III^o^ AH (OR = 26.30, 95% CI = 2.82–245.54). In addition to the adenoid size of the first-born child, from all the analyzed factors that affected the second-born child, only snoring was shown to be a predictive factor of III^o^ AH in the younger sibling (*p* = 0.015, OR = 8.43, 95% CI = 1.51–46.95). The resulting data showed that knowledge of adenoid size in older siblings eliminates the importance of type of tympanogram in the younger child to estimate their risk of III^o^ AH.

Finally, we assess the potential predictive value of snoring in the second-born child by relating it with an A/C ratio of >65% in the first-born child. Over 90% of snoring children whose sibling had confirmed III^o^ AH would later develop III^o^ AH (Table 5). In our series, second-born children in whom snoring occurs and whose older siblings have a known A/C ratio of >65% have about a 46-fold higher risk of III^o^ AH (A/C > 65%) compared to patients who did not meet these two conditions (*p* < 0.001, OR = 46,67, 95% CI = 8.37–260.30) (Table 5).

These results indicate that combining snoring in second-born children with adenoid size in first-born children in the same family clearly improves the prediction of III^o^ AH in second-born patients. 

## 4. Discussion

Our work shows a significant correlation between adenoid size in siblings if an FNE is performed at the same age. Katznelson and Gross observed a difference in the number of adenoidectomies between operated parents and siblings and the control group, which might suggest a familial susceptibility to AH [2]. On the other hand, the authors suggest that parents who were previously operated on or who have a child who was previously operated on might be more willing to allow the surgery to be performed on their second child. Still, our results confirmed the hypothesis of familial susceptibility to hypertrophy based on endoscopically assessed adenoid size. However, Bani-Ata et al. indicated a low significance of tonsillectomy in parental and sibling histories [19], but the size of palatine tonsils is not the main indication for tonsillectomy; therefore, it is difficult to compare a family predisposition to adenoidectomy with tonsillectomy. However, recurrent or chronic inflammation susceptibility of the adenoids or palatine tonsil tissue may lead to chronic activation of the cell-mediated and humoral immune response, which may play a role in hypertrophy [20]. This susceptibility to infection may be caused by genetic dispositions. The role of different variations in inflammatory genetic factors, such as polymorphisms of mannose binding lectin (MBL), toll-like receptors (TLRs), secretoglobulins (SCGBs), or IL-10, were analyzed [20,21,22,23]. Grasso et al. found that the MBL2 00 genotype is a prognostic marker of AH in children [21]. Meanwhile, Babademez et al. stated that *TLR4* polymorphisms were associated with an increased risk of AH, but they did not find the same association when they analyzed *TLR2* polymorphisms [20]. In addition, in the work of Özdaş et al., the presence of single nucleotide polymorphisms (SNPs) of secretoglobulins were associated with an increased risk of AH [22]. Another study demonstrated the role of the *IL-10* genotype GG in resistance to hypertrophy [23]. All these data support the hypothesis that the inheritance of AH is likely polygenic, involving aspects of physiology determined by multiple genes. Moreover, other non-genetic (environmental) factors, such as cytomegalovirus, human herpesvirus type 6, and infections, may play a role in AH [23]. These factors may co-occur in siblings from the same family who are in constant contact with each other. A study performed by Trask et al. shows that both allergic and non-allergic sibling groups showed a larger mean adenoid size on radiographs than controls [24].

Our study offers practical knowledge for pediatricians. Snoring children have a 6-fold greater risk of AH (III^o^ in Bolesławska scale, A/C ratio > 65%) compared to patients who do not snore. In addition, children with an abnormal (not type-A) tympanogram and indirect effusion in the middle ear indicated a six-times greater chance of III^o^ AH compared to children with a type-A tympanogram. The obtained results indicate that second-born children whose older sibling had III^o^ AH have more than a 26-fold greater risk of III^o^ AH compared to patients whose older siblings do not have AH. Second-born children will have a 46-fold increased chance of developing III^o^ AH if they snore and if their older sibling has previously confirmed III^o^ AH. 

AH is the one of the main etiological factors for pediatric sleep disordered breathing (SDB). Lundkvist et al. analyzed parents affected by obstructive sleep apnea (OSAS) and their children, and they concluded that children whose parents were affected by OSAS had a substantially higher risk of hospitalization for SDB [25]. These symptoms were associated with pediatric OSAS or either adenoid or palatine tonsillar hypertrophy. Carmelli et al. analyzed genetic factors in self-reported snoring and excessive daytime sleepiness in twins, arguing that the inheritance of sleep apnea symptoms may be polygenic, but it can also be modulated by the environmental factors in which the twins grow up [26]. The issue of OSAS and hypertrophy of the adenoid and palatine tonsils in siblings was also analyzed by Friberg [27], who showed a significant risk of OSAS in children whose sibling has an OSAS diagnosis, significantly higher than in children with adenoid and palatine tonsils hypertrophy. This study, database research that analyzed AH, was based on a medical diagnosis described in the patient medical history by the ICD-10 code.

In addition, we showed in the whole analyzed group of children a correlation between adenoid size and such adenoid symptoms and related illnesses as rURTI, rhinitis, snoring, poor mucus coverage, and poor tympanometry type. This confirms the role of III^o^ AH in the mentioned factors also described by other authors [1,28,29,30,31].

In our study, we showed a close relationship between AH in children and snoring. We stated that involution of the adenoid in children who snore and who have AH decreases slowly and linearly (Figure 2). The shape of the curve on the graph is similar to that presented by Papaioannou et al. [32]. They analyzed adenoid size in an MRI study in children of different ages and concluded that in children who do not snore, adenoid size increases to 7–8 years of age and then it slowly decreases (parabolic curve), and in the group of children who snore (more than 1 night per week), the reduction in adenoid size occurred slowly until 18 years (linear curve).

In summary, this study shows a great familial correlation between adenoid size in siblings based on real adenoid sizes measured by FNE. Other similar studies were based on a history of performed surgery and reported symptoms or ICD-10 code. Due to a lack of a historical possibility to analyze adenoid size via an endoscopic examination in parents because this technology was unavailable when parents were at their children’s age, only endoscopic images of siblings’ adenoids were comparable.

A limitation in this study was the difficulty of selection of siblings who were examined at the same age, because some parents whose older child was diagnosed with AH or who had presented adenoid symptoms and related illness decided to diagnose their younger child earlier and, based on their own experience earlier, opted for surgery. The influence on sample size is related to the fact that this group was examined by one children’s ENT specialist (A.Z.) in the same ENT outpatient clinic. Further, the repeatability of the tests performed is affected by the use of the same doctor using the same flexible endoscopic system.

## 5. Conclusions

We showed a significant familial correlation between adenoid size in siblings when they reach the same age. The obtained result indicates the environmental and genetic mechanisms of AH, but due to the polygenicity of the issue, more research is necessary.

Our results also suggest that experiences and observations related to the medical history and examination of the older child can be helpful in making a timely diagnosis of the younger child. It is especially important for pediatricians to consider that when an older sibling has a confirmed overgrown adenoid (III^o^ AH) and their younger sibling presents adenoid symptoms, particularly snoring, it is highly probable that they will also have an overgrown adenoid (46 times greater risk).

## Figures and Tables

**Table 1:** Demographic and clinical characteristics of study population according to birth order of siblings.

Characteristic		First-Born Children	Second-Born Children	*p* Value
*n*		49	49	
Age (years)	Mean ± SD	5.0 ± 2.2	5.1 ± 2.2	0.110
	Median (Q25–Q75)	4.3 (3.7–5.6)	4.6 (3.6–5.7)	
Sex	Female	23 (46.9%)	15 (30.6%)	0.134
	Male	26 (53.1%)	34 (69.4%)	
rURTI *	No	14 (28.6%)	24 (49.0%)	0.021
	Yes	35 (71.4%)	25 (51.0%)	
Rhinitis	No	11 (22.4%)	17 (34.7%)	0.210
	Yes	38 (77.6%)	32 (65.3%)	
Snoring	No	17 (34.7%)	13 (26.5%)	0.630
	Occasionally	14 (28.6%)	19 (38.8%)	
	Persistent	18 (36.7%)	17 (34.7%)	
Adenoid size (A/C ratio and (Bolesławska scale, %)	Mean ± SD	63.0 ± 17	59.0 ± 20	0.163
	Median (Q25–Q75)	60.0 (50.0–75.0)	60.0 (50.0–75.0)	
	<35 (B I)	2 (4.1%)	6 (12.2%)	0.396
	35–65 (B II)	27 (55.1%)	25 (51.0%)	
	>65 (B III)	20 (40.8%)	18 (36.7%)	
Adenoid mucus coverage (MASNA scale)	0	15 (30.6%)	14 (28.6%)	0.387
	1	22 (44.9%)	19 (38.8%)	
	2	9 (18.4%)	12 (24.5%)	
	3	3 (6.1%)	4 (8.2%)	
Tympanogram	AA	26 (53.1%)	33 (67.3%)	-
	AB/BA	2 (4.1%)	0 (0.0%)	
	AC/CA	5 (10.2%)	4 (8.2%)	
	BB	10 (20.4%)	7 (14.3%)	
	CB/BC	1 (2.0%)	3 (6.2%)	
	CC	5 (10.2%)	2 (4.1%)	
	A	26 (53.1%)	33 (67.3%)	0.340
	B	13 (26.5%)	10 (20.4%)	
	C	10 (20.4%)	6 (12.2%)	
Thermal season	Summer	21 (42.9%)	27 (55.1%)	0.263
	Winter	28 (57.1%)	22 (44.9%)	

* rURTI—recurrent upper respiratory tract infections.

**Table 2:** Relationships between demographic or clinical factors and adenoid size in children with symptoms suggestive of chronic AH.

Characteristic (*n* = 98)		Adenoid Size (A/C Ratio), %	*p* Value
		Mean ± SD	
Sex	Female	64.1 ± 20.5	0.195
	Male	59.1 ± 17.1	
rURTI	No	50.0 ± 15.9	<0.001
	Yes	68.0 ± 16.7	
Rhinitis	No	51.6 ± 15.6	0.001
	Yes	64.8 ± 18.3	
Snoring	No	50.5 ± 19.3	<0.001
	Occasionally	60.0 ± 15.4	
	Persistent	71.0 ± 15.4	
	No	50.5 ± 19.3	<0.001
	Yes	65.7 ± 16.3	
Adenoid mucus coverage (MASNA scale)	0	52.8 ± 18.2	0.002
	1	60.0 ± 18.1	
	2	70.2 ± 15.8	
	3	73.6 ± 13.5	
Tympanogram	A	54.5 ± 17.8	<0.001
	B	74.6 ± 15.2	
	C	65.6 ± 13.5	
	A	54.5 ± 17.8	<0.001
	Non-A	70.9 ± 15.0	
Thermal season	Summer	58.2 ± 19.8	0.145
	Winter	63.7 ± 17.0	
Place of residence	countryside	59.8 ± 19.9	0.901
	city	60.3 ± 19.4	

**Table 3:** Linear regression analysis for the prediction of adenoid size in children (*n* = 98).

Characteristic	*p* Value	B with 95% CI	Beta (β)
rURTI	0.015	9.04 (1.81–16.27)	0.24
Rhinitis	0.470	2.73 (−4.74–10.20)	0.07
Snoring	<0.001	7.47 (3.94–10.99)	0.33
Adenoid mucus coverage	0.019	3.98 (.67–7.30)	0.19
Tympanogram	0.001	6.25 (2.58–9.92)	0.27

**Table 4:** Logistic regression analysis for the prediction of III^o^ AH (A/C ratio > 65%) in second-born children (*n* = 49).

Characteristic	*p* Value	OR	95% CI
Second-born child factors			
rURTI, yes	0.736	1.37	0.22–8.43
Rhinitis, yes	0.897	1.14	0.15–8.57
Snoring, yes	0.006	6.23	1.7–22.70
Adenoid mucus coverage (MASNA scale), per category	0.14	1.96	0.80–4.79
Tympanogram, non-A	0.029	6.30	1.20–32.99
Second-born child factors and adenoid size of the first-born child			
rURTI, yes	0.543	0.48	0.04–5.15
Rhinitis, yes	0.575	2.05	0.17–25.08
Snoring, yes	0.015	8.43	1.51–46.95
Adenoid mucus coverage (MASNA scale), per category	0.335	1.68	0.59–4.81
Tympanogram, non-A	0.178	4.34	0.51–36.77
Adenoid size of the first-born child, A/C ratio > 65%	0.004	26.30	2.82–245.54

**Table 5:** The relationship between snoring in the second-born child and an A/C ratio of >65% in the first-born child and an A/C ratio > 65% in the second-born children group with symptoms suggestive of chronic AH.

		Adenoid Size (A/C Ratio, %) of the Second-Born Child		*p* Value	OR (95% CI)
		≤65	>65		
Snoring in the second-born child and A/C ratio > 65% in the first-born child	yes	28 (90.3%)	3 (16.7%)	<0.001	46.67 (8.37–260.30)
	no	3 (9.7%)	15 (83.3%)		

## Data Availability

Additional data supporting reported results may be available for request.
